# Statistical methods for evaluating the fine needle aspiration cytology procedure in breast cancer diagnosis

**DOI:** 10.1186/s12874-022-01506-y

**Published:** 2022-02-06

**Authors:** Carolla El Chamieh, Philippe Vielh, Sylvie Chevret

**Affiliations:** 1 Department of Biostatistics and Medical Information, INSERM UMR1153 ECSTRRA Team, Hôpital Saint Louis, AP-HP, Paris, France; 2 Medipath & American Hospital of Paris, Paris, France

**Keywords:** Breast cancer, Diagnosis, Verification bias, Suspect results, Fine needle aspiration cytology

## Abstract

**Background:**

Statistical issues present while evaluating a diagnostic procedure for breast cancer are non rare but often ignored, leading to biased results. We aimed to evaluate the diagnostic accuracy of the fine needle aspiration cytology(FNAC), a minimally invasive and rapid technique potentially used as a rule-in or rule-out test, handling its statistical issues: suspect test results and verification bias.

**Methods:**

We applied different statistical methods to handle suspect results by defining conditional estimates. When considering a partial verification bias, Begg and Greenes method and multivariate imputation by chained equations were applied, however, and a Bayesian approach with respect to each gold standard was used when considering a differential verification bias. At last, we extended the Begg and Greenes method to be applied conditionally on the suspect results.

**Results:**

The specificity of the FNAC test above 94%, was always higher than its sensitivity regardless of the proposed method. All positive likelihood ratios were higher than 10, with variations among methods. The positive and negative yields were high, defining precise discriminating properties of the test.

**Conclusion:**

The FNAC test is more likely to be used as a rule-in test for diagnosing breast cancer. Our results contributed in advancing our knowledge regarding the performance of FNAC test and the methods to be applied for its evaluation.

**Supplementary Information:**

The online version contains supplementary material available at (10.1186/s12874-022-01506-y).

## Background

Worldwide, breast cancer is considered the most prevalent cancer among women and the second most common cancer overall [1]. According to the *American Institute for Cancer Research*, France was classified in 2018 as having the fourth highest prevalence of breast cancer worldwide after Belgium, Luxembourg, and the Netherlands [2]. Approximately 59,000 women are diagnosed with breast cancer yearly in France according to *Santé Publique France* [3].

When diagnosing breast tumors, the Fine Needle Aspiration Cytology (FNAC) test is widely used [4]. It has been reported as a simple, minimally invasive, and cost-effective technique for the diagnosis of breast cancer [5]. However, whether it could be used as a rule-out or rule-in test is a matter of concern to be evaluated. A rule-out test (also known as triage test) is characterized by a higher sensitivity (*Se*) than specificity (*Sp*), establishing the absence of disease when its result is negative, and requiring the use of further testing to confirm the presence of the disease when its result is positive. A rule-in test is characterized by a higher *Sp*, to confirm the disease (rule-in) when its result is positive [6–9]. Decisions for rule-in or rule-out could also be based on likelihood ratios (LR), so that the diagnostic test can lead to the highest disease probability for a positive test result (rule-in) or the lowest disease probability for a negative test result (rule-out). Thus, to assess the clinical utility of the diagnostic test, the *Se*, *Sp*, disease probability, and *LR* must be taken into account [8].

In breast cancer diagnosis, histology is the worldwide recognized gold standard that is performed in case of positive findings from either clinical, imaging, or FNAC results (resulting in a “triple test” approach). Consequently, only a non-representative sub-sample of the original study subjects will benefit from the histology gold standard [4, 10], leading to partial verification bias [11]. Moreover, an alternative gold standard, that is the long-term (18 months) follow-up of breast imaging findings, is usually performed for the rest of the participants, defining a differential verification bias, given that such a measure of disease status is less accurate than histology [11–13].

In many cases, the results from a given diagnostic test do not exclusively fall into the “positive” and “negative” categories. This was notably the case with the FNAC test. Clear report and analysis of such indeterminate (inconclusive) test results are needed in order to avoid bias in the estimation of the test performance [14].

The objective of this study was to evaluate the interest of the FNAC in the diagnostic strategy of breast cancer, using methods that allow handling these statistical issues.

To evaluate the performance of the FNAC test, we used data collected from a retrospective observational study, that included all patients consecutively seen at the Gustave Roussy Institute.

## Methods

A total of 1 740 women with 1 820 breast tumors were included between April 2004 and March 2007. In addition to the FNAC, subjects’ imaging findings (mammography and ultrasound) were evaluated for breast cancer diagnosis, with a classification of the risk of breast cancer based on the American College of Radiology’s (ACR) guidelines. Cytopathologic, and histopathologic results were extracted from the hospital’s computerized medical records.

Table 1 summarizes the results of the FNAC test and of the two standards used to assess the existence of breast cancer (*D*^+^) or not (*D*^−^). Note that these figures refer to tumor samples (not to patients). Indeed, according to the study oncologist, we considered and analyzed the 1 820 breast tumors altogether, assuming independence between the observations of the subjects with more than one tumor. It is also noteworthy that some exceptions of the diagnostic strategy were observed (with 38 patients having positive FNAC tests but not verified by histology).

**Table 1:** Data presenting the results of FNAC test compared to histology and follow-up gold standards

Gold standard	Histology		Follow-up		Lost to follow-up	Total
FNAC*	*D*^+^	*D*^−^	*D*^+^	*D*^−^		
Positive	803	1	12	0	26	842
Suspect	120	31	0	1	2	154
Negative	24	115	1	471	160	771
Insufficient	18	26	0	6	3	53
Total	965	173	13	478	191	1820

*FNAC fine needle aspiration cytology

Cytologic diagnoses were classified into four categories: benign, suspect, malignant, and insufficient. Suspect results were defined as neither positive nor negative results, that is where the cytologist could not affirm nor refute the malignancy, though the latter being highly suspect of malignancy [5, 15]. Insufficient results were those achieved due to insufficient materials. Indeed, due to sampling technical issues, the FNAC test may have yielded insufficient cellularity. Since the obtained material was insufficient to be tested, no definitive diagnosis could be done, resulting in missing data. However, according to experts from the field, such data could not be combined with suspect results, but considered completely missing at random (MCAR). Therefore, the 53 samples with insufficient materials of the FNAC test were excluded from further analyses.

Data presented in Table 1 holds some statistical issues that should be taken into consideration in the analysis.

### Handling suspect diagnostic test results

The first issue refers to the recorded responses of the FNAC test. Indeed, while a diagnostic test usually yields binary responses, the FNAC test is a 3-valued outcome measure, that includes suspect results in addition to positive and negative test results. These latter outcomes (*n*=154) could be defined and treated as a non-positive, non-negative results [16].

For the gold standards, we first ignored its source, pooling results from the histology and the follow-up, and excluding missing data (lost to follow-up). Accordingly, data can be described using a 3×2 decision matrix (Table 2).

**Table 2:** Decision matrix for handling suspect results

FNAC*	Gold standard	
	Disease (*D*^+^)	No disease(*D*^−^)
Positive (*T*^+^)	*a*	*b*
Suspect (T ±)	*e*	*f*
Negative (*T*^−^)	*c*	*d*

*FNAC fine needle aspiration cytology

Several strategies were used.

#### Estimates of performance measures based on a 2×2 cell matrix

The simplest approach consisted in resuming the data in a 2×2 cell matrix, which allows applying the usual diagnostic measures estimators directly. This required to combine the suspect results with one of the positive or negative values of the FNAC. Four approaches were considered.

The “conventional” strategy consisted in excluding suspect results from the calculation [16]. In the “worst case”, the suspect results were combined with negative results in diseased patients and with the positive ones in non-diseased participants [16]. In the “best case”, conversely, the suspect test results were considered as positive in diseased participants, and as negative in non-diseased [5, 16]. At last, we applied Multivariate Imputation by Chained Equations (MICE) to impute those suspect results, assuming missing at random mechanisms (MAR) [17, 18]. Given the rate of such missing data, M = 10 complete datasets were imputed, where the imputation model included all the factors possibly impacting the presence of the disease (patient’s age, lesion location within the breast, tumor size, side of the breast tumor, and ACR), results of FNAC, histology, and follow-up. Then, from each of these tables, estimates (except for LR) of the diagnostic performance of the cytology test with their intra-imputation variance were pooled by using Rubin’s Rule [17]. We then calculated the corresponding 95% confidence interval of each estimate [18].

#### Estimates of performance measures based on a 3×2 cell matrix

In contrast with the previous approaches, we secondly tried to respect the data structure of the 3×2 matrix.

Simel et al. [16] proposed conditional definitions of diagnostic performance measures, conditioned on the positive or negative test results, so-called positive or negative “test yield” (*Y*^+^,*Y*^−^):

$$Y^{+} = P(T^{+} \cup T^{-}|D^{+})= \frac{a+c}{a+e+c} $$

and

$$Y^{-} = P(T^{+} \cup T^{-}|D^{-})= \frac{b+d}{b+f+d} $$

Conditional measures of sensitivity (*Se*^*c*^) and specificity (*Spe*^*c*^) were defined, resulting in similar estimators as those of the “conventional strategy” described above [16]:

$$Se^{c}=\frac{P(T^{+}|D^{+})}{P(T^{+} \cup T^{-}|D^{+})}=\frac{a}{a+c} $$

$$Spe^{c}=\frac{P(T^{-}|D^{-})}{P(T^{+} \cup T^{-}|D^{-})}=\frac{d}{b+d} $$

Simel et al. [16] and Eusebi et al. [19] also defined the *conditional LR of suspect results* (*LR*±), *the overall test yield*, and the test *accuracy* of the test, as follows:

1 $$ LR\pm = \frac{P(T\pm |D^{+})}{P(T\pm |D^{-})} = \frac{1-(Y^{+})}{1-(Y^{-})}   $$

2 $$ \textrm{Overall test yield} = \frac{a+b+c+d}{a+b+c+d+e+f}   $$

3 $$ Accuracy= \frac{a+d}{a+b+c+d+e+f}   $$

Exact 95% confidence interval (95% CI) of *Se*, *Sp*, test yields, and accuracy, were estimated. We used the Simel et al. 95% CI formula for the positive and negative *LR* (*LR*^+^ and *LR*^−^) [16, 20].

### Handling verification bias in gold standard

In the previous sections, we ignored the different sources of the gold standard, that is, assuming that disease status was similarly measured at the same time as the FNAC for all subjects. The estimates of the 2 x 2 matrix will be considered as naive estimates in the further analyses, since they did not take into account the presence of verification bias. However, the disease status was not always measured by histology, but only when the “triple test” provided positive findings. Otherwise, diagnosis was based on follow-up imaging of the breast. Moreover, there were missing data in the verification procedure (lost to follow-up, *n*=191). We thus applied methods to handle this verification bias.

#### Partial verification bias

First, we considered the partial verification bias, which is, treating histology as the only gold standard for diagnosis measure, so that patients not verified by histology (either with or without follow-up) had missing disease status.

##### Begg and Greenes method

Begg and Greenes proposed to infer about the probabilities of test results (*T*) given the disease status (*D*), in the presence of missing disease status, that is, when there is only a subset of patients whose disease status has been completed (*V*=1).

Let *X* be the vector capturing all the other information likely to influence the selection of *V*. In our setting, it represents the imaging and clinical information. Although the disease process affects both *T* and *X*, it only affects selection (*V*) through its influence on *T* and *X*. Thus, given that conditional independence between the verification status *V* and *D*, the probability of *T* given *D* and *X* is defined by:

$$P(T|D,X)=\frac{P(T,X) P(D|T,X,V=1)}{\sum_{T} P(T,X) P(D|T,X,V=1)} $$

They proposed to estimate the non verified patients by applying inverse weighting, using the observed proportions of diseased and non-diseased among the verified patients by histology (*V*=1) to calculate the expected number of diseased and non-diseased patients among non-verified patients (follow-up or lost to follow-up), as reported in Table 3. Accuracy measures were then computed as if all disease status had been measured by histology [11].

**Table 3:** Begg and Greenes correction method

		*D*^+^	*D*^−^	Total
*V*=1(histology)	*T*^+^	*a*	*b*	
	*T*^−^	*c*	*d*	
*V*=0 (non verified)	*T*^+^	*a*^′^	*b*^′^	$T_{0}^{+}$
	*T*^−^	*c*^′^	*d*^′^	$T_{0}^{-}$

where $a' = a/(a + b)\times T_{0}^{+}$;

$b' = b/(a + b)\times T_{0}^{+}$;

$c' = c/(c + d)\times T_{0}^{-}$;

$d' = d/(c + d)\times T_{0}^{-}$

We applied the method on the “conventional strategy” described above. We combined the verified with non-verified patients as if all of them had been verified by histology [11], applied the adjusted *Se* = (*a*+*a*^′^)/(*a*+*a*^′^+*c*+*c*^′^) adjusted *Sp* = (*d*+*d*^′^)/(*d*+*d*^′^+*b*+*b*^′^), and derived the *LR*^+^ and *LR*^−^.

##### MICE

Given that the verification by histology depends on patients’ observed data, missing gold standard could be considered as missing at random (MAR). Thus, multiple imputation by chained equations (MICE) was applied [11, 21, 22], and compared to the Begg and Greenes method. It was applied to the conventional strategy of naive estimates. Missing data of unverified patients (with follow-up or not) was imputed with *M*=38 complete tables, given the percentage of missing data in this sample. The imputation model included all the factors likely to impact the presence of the disease (patient’s age, lesion location within the breast, size of the tumor, side of the breast tumor, and ACR), results of FNAC, and histology. Estimates of *Se* and *Sp* of each of the *M* analyses were then combined using Rubin’s rule to produce the estimate and confidence interval that incorporate between and within imputation variability [23]. We could then estimate the *LR*^+^ and *LR*^−^.

#### Differential verification bias

Second, we corrected for the differential verification bias, considering “follow-up” as an alternative gold standard to histology.

Due to the imperfect nature of follow-up, the estimated *Se* and *Sp* may be incorrect [10]. A Bayesian correction approach [12] was applied to the conventional strategy. First, patients lost to follow-up were excluded. Second, they were imputed by applying MICE [21]. The information from the observed data was summarized into a likelihood function, defined as a product of four independent binomial density functions, each corresponding to the probability of a positive result on a gold standard (*D*^+^) conditional on the index test (FNAC) (*T*)[12]:

4 $$ {}P(D^{+}|T^{+})\times (1-P(D^{+}|T^{+}))\times P(D^{+}|T^{-})\times (1-P(D^{+}|T^{-}))  $$

with

5 $$ {}{\begin{aligned} P(D^{+}|T^{+}) &= sD{\frac{prev\times sT}{(prev \times sT) +(1-prev)(1-cT)}}\\ &\quad+(1-cD)\frac{(1-prev)(1-cT)}{(prev\times sT) +(1-prev)(1-cT)} \end{aligned}}  $$

And

6 $$ {}{\begin{aligned} P(D^{+}|T^{-}) &= sD{\frac{prev\times(1-sT)}{(prev\times(1-sT))+(1-prev)cT}}\\&\quad +(1-cD)\frac{(1-prev) cT}{(prev\times(1-sT))+(1-prev)cT} \end{aligned}}  $$

where:

- *sT*,*cT*: sensitivity, specificity of FNAC,
- *sD*,*cD*: sensitivity, specificity of histology or follow-up,
- *prev*: prevalence of the disease.

These formulas were applied for each of the histology and follow-up gold standards. Bayesian inference was applied, where *sT*, *cT*, *sD*, *cD*, and *prev*, were considered as random variables with prior distributions. According to deGroot et al, we used independent Beta (*α*,*β*) prior distributions [12]. Given that the histology reference is the perfect gold standard for breast cancer diagnosis, its *Se* and *Sp* were set at 1 [24, 25]. We used informative prior distribution Beta (172.55, 30.45) for both *Se* and *Sp* of imaging follow-up, corresponding to a density centered at 0.85 with estimated standard deviation derived from 1/4 of the range, 0.80-0.90 [12]. We used non informative Beta (1,1) priors for *sT*,*cT*,*prev*, to limit the incorporation of any subjective prior opinion [12].

Using Jags software, the likelihood function was combined with the prior using Bayes theorem to derive posterior distribution. We ran a total of 20 000 iterations, of which we dropped the first 2 000 to allow for a burn-in period. The convergence of the Markov Chain Monte Carlo was checked and summary statistics (posterior mean, 2.5% and 97.5% quantiles) of the parameters of interest were computed.

We checked the effect of the priors chosen by a sensitivity analysis (see Additional file 1).

### Handling both suspect test results and verification bias

At last, we aimed to handle both statistical issues (suspect results and verification bias) in evaluating the performance of the FNAC test.

We proposed to apply the Begg and Greenes method to the 3×2 matrix that estimated test characteristics conditionally to the suspect results. Disease status was only based on histology, and all the other patients (followed-up or lost to follow-up) were considered as non-verified. We extended formulas used to estimate the results of non-verified patients, in order to estimate their suspect results (*e*^′^ and *f*^′^), as reported in Table 4.

**Table 4:** Begg and Greenes correction method for the 3*×*2matrix

		*D*^+^	*D*^−^	Total
*V*=1 (histology)	*T*^+^	*a*	*b*	
	T ±	e	f	
	*T*^−^	*c*	*d*	
*V*=0 (non verified)	*T*^+^	*a*^′^	*b*^′^	*T*0+
	*T*±	e’	f’	*T*0±
	*T*^−^	*c*^′^	*d*^′^	*T*0−

where *e*^′^=*e*/(*e*+*f*)×*T*0±

and *f*^′^=*f*/(*e*+*f*)×*T*0±

We estimated the adjusted *Se*, *Sp* from the combination of verified and non-verified patients results, and derived the *Y*^+^,*Y*^−^ and the *LR*±, by applying the conditional measures defined in the section namerefsuspec.

### Computation

For data description, continuous variables were presented as mean (standard deviation), and categorical variables as frequency (percentage). The diagnostic performance measures of the FNAC were presented by the point estimate with 95% confidence interval, or by the posterior mean with 95% credible intervals when the Bayesian approach was applied.

Analyses were performed using the statistical software R, version 4.0.4 (https://cran.r-project.org/).

## Results

**Fig. 1:**
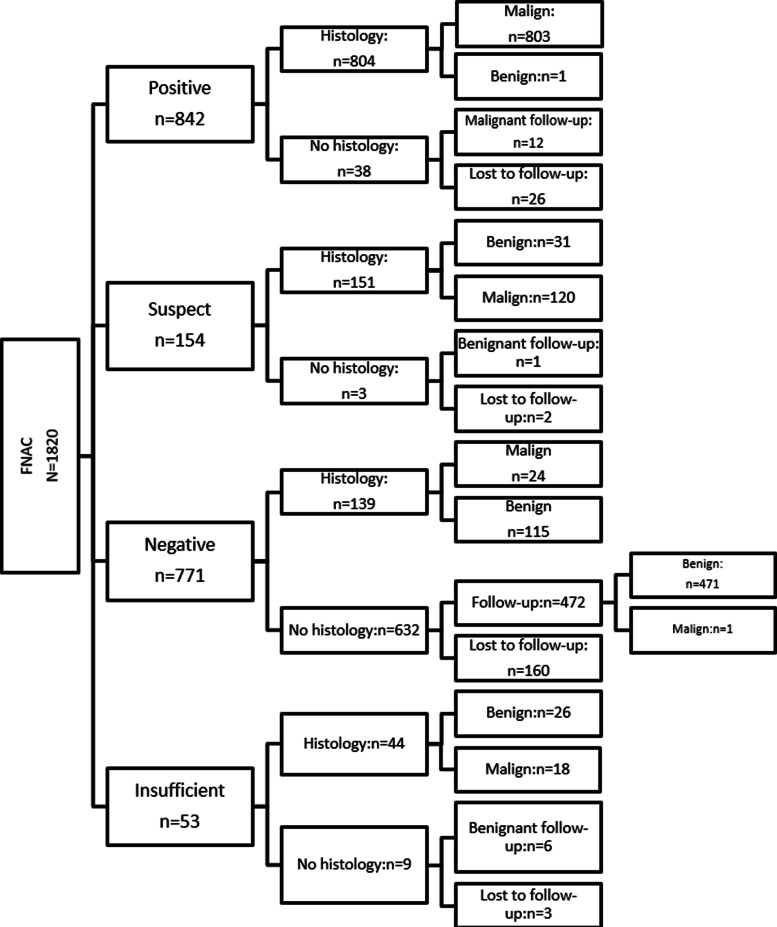
Flow Chart of the study

The flow chart of the study is reported in Fig. 1.

Table 5 summarizes participants’ characteristics and disease status according to the FNAC results. Most of the subjects with positive (77%) and suspect results (51%) had a breast imaging coded by an ACR of 5 or 4 (21% and 41% for positive and suspect results, respectively). Conversely, most of the participants with negative results had an ACR of 3. Concerning the disease status, most of the patients having positive or suspect FNAC results were verified by histology proving a malignant tumor status (95.5% and 78% respectively), and most of the participants with negative FNAC results had a benign histological status (61%). Lost to follow-up patients were mainly those with negative FNAC results (21%).

**Table 5:** Description of the results according to the FNAC* results

Characteristic	Negative (N = 771)^a^	Positive (N = 842)^a^	Suspect (N=154)^a^
Age (years)	50 (13)	61 (14)	57 (13)
Side			
Right	373 (48%)	383 (45%)	71 (46%)
Left	398 (52%)	459 (55%)	83 (54%)
Size (mm)	15 (37)	28 (41)	17 (15)
ACR*			
1	4 (1%)	1 (0.1%)	0 (0%)
2	97 (12.5%)	3 (0.4%)	0 (0%)
3	440 (57%)	13 (1.5%)	12 (8%)
4	188 (24.5%)	177 (21%)	62 (41%)
5	39 (5%)	643 (77%)	78 (51%)
Unknown	3	5	2
Echoguided	626 (81%)	342 (41%)	96 (62%)
Infiltrating canal carcinoma	22 (2.9%)	707 (84%)	98 (64%)
Unknown	2	0	0
Intracanalar carcinoma	8 (1.0%)	161 (19%)	33 (21%)
Infiltrating lobular carcinoma	9 (1.2%)	95 (11%)	20 (13%)
Lobular carcinoma in situ	5 (0.6%)	17 (2.0%)	8 (5.2%)
Disease status			
Benign histology	115 (15%)	1 (0.1%)	31 (20%)
Malignant histology	24 (2.9%)	803 (95.5%)	120 (78%)
Benign follow-up	471 (61%)	0 (0%)	1 (0.5%)
Malignant follow-up	1 (0.1%)	12 (1.4%)	0 (0%)
Lost to follow-up	160 (21%)	26 (3%)	2 (1.5%)

*FNAC fine needle aspiration cytology, *ACR american college of radiology

^a^Mean (SD); n (%)

### Handling suspect diagnostic test results

The standard diagnostic measures of the 2×2 cell matrix are presented in Table 6. As expected, the *Se*, *Sp*, *LR*^+^ and *LR*^−^ of the conventional strategy ranged between the worst and best cases, with values higher than those provided by MICE.

**Table 6:** Different estimates [with 95% CI] of FNAC test performance according to the different methods

Handling verification bias	Without					With (Begg and Greenes)
Cell matrix			2×2		3×2	3×2
Methods	Worst case	Conventional	Best case	MICE	Conditional	Conditional
*Se*/ *Se*^*c*^	0.848	0.970	0.974	0.946	0.970	0.863
	[0.82-0.87]	[0.96-0.98]	[0.96-0.98]	[0.93-0.96]	[0.96-0.98]	[0.84-0.884]
*Sp*/ *Spe*^*c*^	0.946	0.998	0.998	0.986	0.998	0.998
	[0.93-0.96]	[0.99-0.99]	[0.99-0.99]	[0.98-0.99]	[0.99-0.99]	[0.991-0.999]
*LR*^+^	16	570	603	67.5	570	552
	[11.4-22.2]	[80.4-4,037]	[85-4,273]	[-]	[80.4-4,037]	[78-3911.5]
*LR*^−^	0.160	0.029	0.026	0.055	0.029	0.137
	[0.14-0.19]	[0.02-0.04]	[0.02-0.04]	[-]	[0.02-0.04]	[0.117-0.160]
Accuracy	0.887	0.982	0.984	0.962	0.887	0.837
	[0.87-0.90]	[0.97-0.99]	[0.98-0.99]	[0.95-0.97]	[0.871-0.902]	[0.819-0.854]
prev	0.608	0.588	0.608	0.608	0.608	0.620
	[0.583-0.632]	[0.561-0.612]	[0.583-0.632]	[0.583-0.632]	[0.583-0.632]	[0.597-0.643]
*Y*^+^	-	-	-	-	0.875	0.889
					[0.852-0.895]	[0.868-0.907]
*Y*^−^	-	-	-	-	0.948	0.952
					[0.928-0.964]	[0.933-0.967]
LR ±	-	-	-	-	2.4	2.5
					[1.7-3.5]	[1.5-3.5]

*Se* sensitivity, *Sp* specificity, *Se*^*c*^ conditional sensitivity, *Spe*^*c*^ conditional specificity, *LR* likelihood ratio, *prev* disease prevalence, *Y* test yield

As reported above, using the 3×2 cell matrix only provided different estimates of test accuracy and of disease frequency than the naive conventional strategy. Moreover, the test yields could be computed, *Y*^+^= 0.875; *Y*^−^= 0.948; overall test yield = 0.903 [0.888-0.918]; and *LR*± = 2.4 (Table 6).

### Handling verification bias in gold standard

**Fig. 2:**
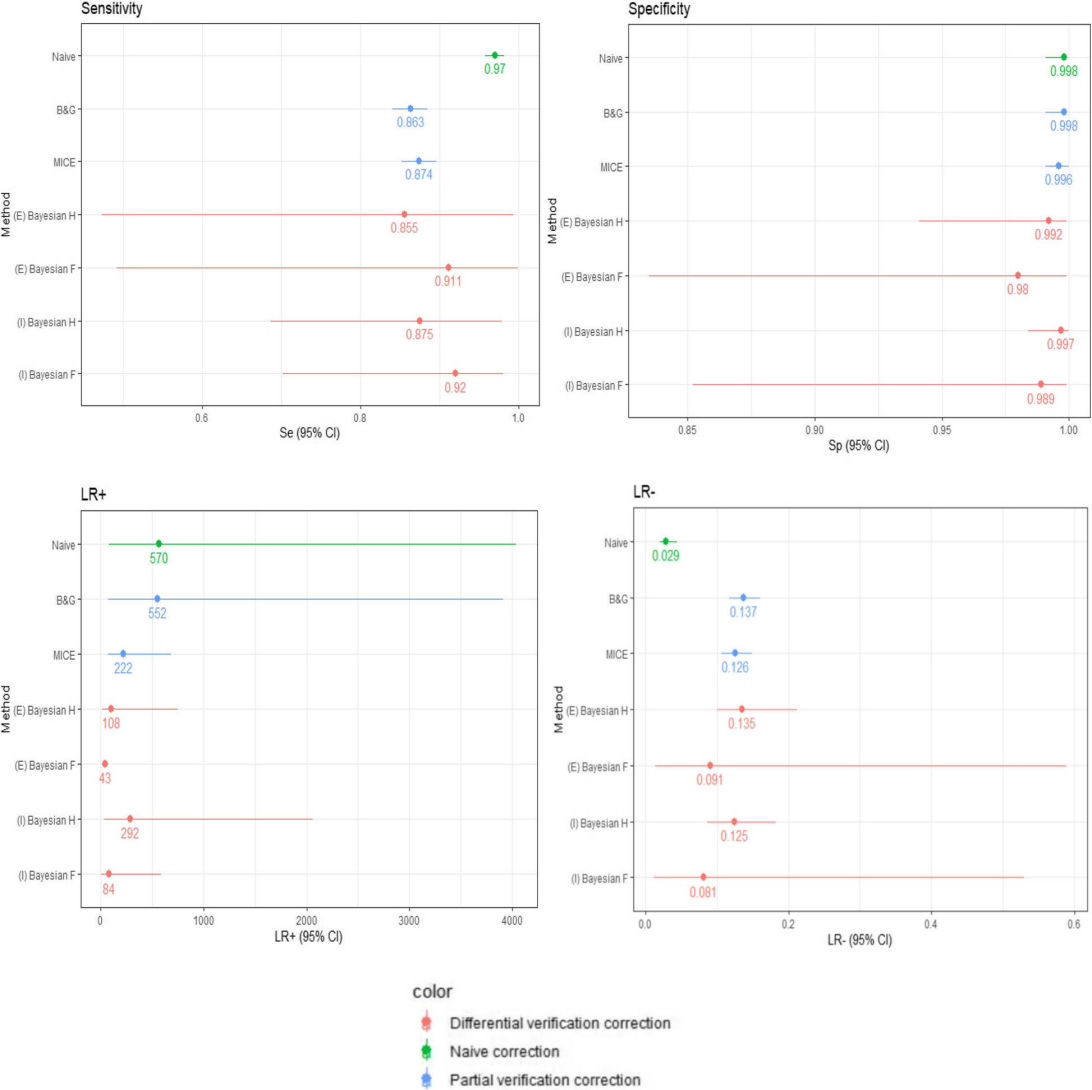
Different methods correcting for verification bias. For the Begg and Greenes method, missing data of non-verified patients were computed, with a= 38, b= 0, c= 109 and d= 523. Legend: (E) Bayesian: Bayesian method excluding patients lost to follow-up (NA) ; (I) Bayesian: Bayesian method imputing NA; H: withrespect to histology; F: with respect to follow-up

Figure 2 presents estimates reached from the different methods, compared to the naive conventional strategy that did not take into account the verification bias. All the methods in Fig. 2 were applied on the conventional strategy (excluding suspect results).

When handling partial verification bias, estimates of *Se* and *LR*^+^ were the most impacted, while those of *Sp* and *LR*^−^ were poorly affected. Actually, the *Se* decreased from 97% to 86% with Begg and Greenes and to 87% with MICE, while effects on *Sp* were slight (with estimates ranging from 99.8% when ignoring this source of bias down to 99.6% with MICE). Estimations from Begg and Greenes and MICE were close. When applying the Bayesian approach, the *Se* with respect to histology either when excluding lost to follow-up (NA) or imputing them was the lowest (0.855 and 0.875), compared to the one with respect to follow-up and to the naive estimate, and very close to the values of partial verification correction methods.

By contrast, the *LR*^+^ decreased from 570 to 222 (that is, a 61% decrease) with MICE and *LR*^−^ increased from 0.029 to 0.137 (that is, a 3.72 fold increase) with Begg and Greenes for *LR*^−^. When applying the Bayesian approach, the *LR*^+^, and *LR*^−^ with respect to follow-up were the lowest among all the methods (except for the naive estimate having the lowest *LR*^−^). There were minimal differences between excluding NA and imputing them when using the Bayesian approach for all the tested parameters.

### Handling both suspect test results and verification bias

When applying Begg and Greenes on the 3×2 matrix to handle suspect results (with histology as the only gold standard), we completed missing data of non-verified patients and obtained: *e*^′^ = 2 and *f*^′^=1. Then we estimated the adjusted conditional measures and yielded the same *Se*^*c*^,*Spe*^*c*^,*LR*^+^ and *LR*^−^ than those obtained when applying the Begg and Greenes on the conventional strategy (Fig. 2).

In addition, we estimated the *Y*^+^ = 0.889, *Y*^−^ = 0.952 and *LR*± = 2.5, that were close to those obtained when applying the 3×2 matrix that handled only the suspect test result and neglected the verification bias (Table 6).

In all presented results from different proposed methods, the *Sp* was always higher than *Se* with minimal variation between methods. The *LR*^+^ estimates depended on the method, varying from 16 to 603 between methods, with very large 95% CI.

## Discussion

To our knowledge, this is the first study to be applying methods for correcting the major statistical issues encountered while evaluating the FNAC test in diagnosing breast cancer. These issues, namely suspect results and verification bias, are common in diagnostic research settings [26]. They should be reported in the data analysis and treated in order to avoid biased estimations of the test characteristics. Therefore, we focused on providing unbiased estimates of diagnostic test sensitivity, specificity, positive and negative test yields, and positive, negative, and conditional likelihood ratios, using methods previously proposed to handle such data issues.

First, the suspect results had to be taken into account. The general description of the patients according to the FNAC results (Table 5) shows that the characteristics of women with suspect tests are not always similar to those of women with positive tests. In some cases, they could be closer to those of negative results, thus, preventing a combination of the suspect results with the positive ones in all cases. This explains the way of combination of the suspect results with the positive or negative ones applied in the 2×2 matrix. However, forcing suspect results into negative or positive cells applied in the worst and best case of the 2×2 matrix may lead to biased estimations. The 3×2 cell matrix summarizes all the data observed including suspect results, giving more characteristics to the diagnostic test such as *Y*^+^,*Y*^−^ and LR ±. Note that the *Se*^*c*^ and *Spe*^*c*^ obtained when we only handled the suspect results, applying the conditional 3×2 cell matrix proposed by Simel et al. [16], were close to those obtained by Sustova *et al* [4]. However, verification bias had to be handled too.

To correct partial verification bias and assuming a MAR mechanism, we used the Begg and Greenes method, then MICE, as applied by several previous studies [11*,*^22^*,*^27^*–*29*]. De Groot et al.[*11*] concluded that both methods could be used when missing mechanisms are known, though multiple imputation could be used otherwise. Other studies [*13*,*^27^*] used different methods such as an empirical Bayesian approach with Beta prior for test characteristics estimates, and the maximum likelihood estimates given by the expectation–maximization algorithm by Kosinski and Barnhart [*30*], when the mechanism is missing not at random (MNAR), that is, when missing data depended on unrecorded information related to the disease status [*27].

In our data, a verification by follow-up was further introduced as an alternative reference for diagnosing breast cancer, defining a differential verification bias, not to be confused with partial disease verification. Neglecting this differential verification will overestimate the *Se* and *Sp* explaining the higher estimates in Table 6. Results were reported with respect to each gold standard separately, to provide informative and unbiased measures of accuracy, as previously presented in published studies [10*,*^12^].

In this study, suspect test results, and verification bias were both present. Therefore, a correction targeting both issues had to be implemented. We thus extended the Begg and Greenes method to handle suspect results. Our results were in the range of previous reports. The *Se* was estimated at 0.863 which is lower than the previously reported ones [4,15], and slightly higher than the *Se* of 0.83 reported by Nemer [31]. The *Sp* was estimated at 0.998, equal to that obtained by Sustova et al. [4*], higher than that reported by Farras et al.(0.908) [*15*], and non markedly lower than the 100% reported by Nemer [*31*]. Regarding test yields, they were defined and used by Simel et al. [*16], who concluded with the need to incorporate them into the operating characteristics, to assess the probability of obtaining useful and exact results. Consequently, if the test is expansive and risky, with a low test yield, the test would not be obtained. Due to the very low frequency of non-verified patients having suspect results (*n*=3), the *Y*^+^ and *Y*^−^ were very close to those obtained with the 3×2 matrix that neglected the verification bias. We reported high rates of 88% and 95% respectively, indicating a high probability of obtaining positive or negative results when disease is absent or present, with a higher probability with the absence of the disease, thus, the test has a low probability to yield non-positive, non-negative results. No prior studies reporting these values were found in the literature. The precision of the estimations was illustrated by the 95% CI that was narrow.

All the *Se*, *Sp*, *Y*^+^ and *Y*^−^,*LR*^+^ and *LR*^−^ estimated in our study showed that the FNAC test could be a reliable method for differentiating benign from malignant masses. Indeed, regardless of the used method, all the results vary in a way that labels the FNAC as a rule-in test. *Sp* of FNAC was always higher than its *Se*, with minimal variation between methods and a narrow 95% CI indicating precise estimations. As well, the *LR*^+^ was always greater than 10 indicating important FNAC properties to yield a positive result in diseased patients. But, the *LR*^+^ was the most dependent parameter with very different values varying between methods, and with large 95% CI, leading to imprecision. Moreover, such a position (rule-in test) could be related to the fact that women benefited first from clinical and imaging testing.

Some limitations of our work should be considered. First, we proposed a method that takes into account both issues of suspect results and verification bias, however we only corrected for a partial verification bias, while a differential verification was also present in the data collected. Our method could be extended to apply the Bayesian approach in correcting the differential verification bias on a 3×2 matrix, by defining a likelihood function and priors of *Y*^+^ and *Y*^−^. Second, a simulation study could take place in further researches to confirm the ideal fitted method to be applied when evaluating such a diagnostic procedure. Moreover, many other statistical issues were not treated in this paper. As above mentioned, subjects in this retrospective study also benefited from clinical and imaging findings, that were not taken specifically into consideration when analyzing FNAC characteristics due to lack of accessibility to the clinical data. Further studies evaluating the importance of integrating FNAC in combination with clinical and imaging data (triple test) for the improvement of diagnostic performance should be conducted [5]. Furthermore, women for whom the histological diagnosis was not initially performed, diagnosis was based on the disease status evolution in the upcoming 18 months of follow-up. This will further result in using potentially, at the time of the FNAC test, a prediction, rather than a diagnosis, due to the time-lag between the cytology test and the gold standard. This needs to be taken into account in order to avoid biased estimations of the test performance. Last, note that we used a four-class system of cotation for the FNAC since the study was scheduled at Gustave Roussy cancer center in 2014, that was before the proposal of a five-category classification (insufficient, benign, atypical, suspicious, malignant) published by Vielh et al. in 2017 [32], and internationally recommended under the auspices of the International Academy of Cytology in 2019 [33]. Further work should report and analyze indeterminate (inconclusive) test results with the use of the newly proposed 5-class system. This will allow the reevaluation of the FNAC performance by distinguishing atypical and suspicious among the indeterminate category. Despite the study limitations, our results contributed in advancing our knowledge regarding the performance of FNAC test and the methods to be applied for the evaluation.

In conclusion, FNAC is widely used in the diagnostic strategy of breast cancer. The present study shows the variability of resulted estimations among the proposed methods, though the specificity of the FNAC test was always higher than its sensitivity suggesting the use of FNAC as a rule-in test, that highly indicates a malignancy if positive. Future clinical studies should be encouraged to evaluate and validate this test’s characteristic. Insufficient results due to technical issues or inconclusive findings are often ignored in many studies in order to fit the standard approach based on a 2x2 matrix. As the histology is an expensive and invasive test, it is exclusively indicated when “triple test” finding is positive, making the verification bias unavoidable. All these statistical issues should be clearly reported and handled in the analysis of any future clinical study aiming at evaluating this diagnostic test in other settings. Ideally, researchers should avoid partial and differential verification when conducting a diagnostic study. Nevertheless, if unpreventable, data should be analyzed separately for each gold standard [30], and researchers should clearly discuss the potential clinical consequences.

## Supplementary Information

**Additional file 1** Figure legend: (E): excluding patients lost to follow-up; H: with respect to histology; F: with respect to follow-up; (1): non-informative beta priors for FNAC, informative prior distribution (Beta (172.55, 30.45)) for both Se and Sp of imaging; (2): non-informative beta priors for FNAC and for imaging with respect to follow-up; (3): informative priors for the Se and Sp of FNAC (beta(525,55.1) and beta(465,56)respectively), informative priors for imaging as used in (1)

## Data Availability

The dataset used and/or analyzed during the current study are available from the corresponding author on reasonable request.
