# Supplementary figures and images for: Statistical methods for evaluating the fine needle aspiration cytology procedure in breast cancer diagnosis
Source: BMC Med Res Methodol. 2022 Feb 6;22:40. doi: 10.1186/s12874-022-01506-y (PMC8818244; doi:10.1186/s12874-022-01506-y)

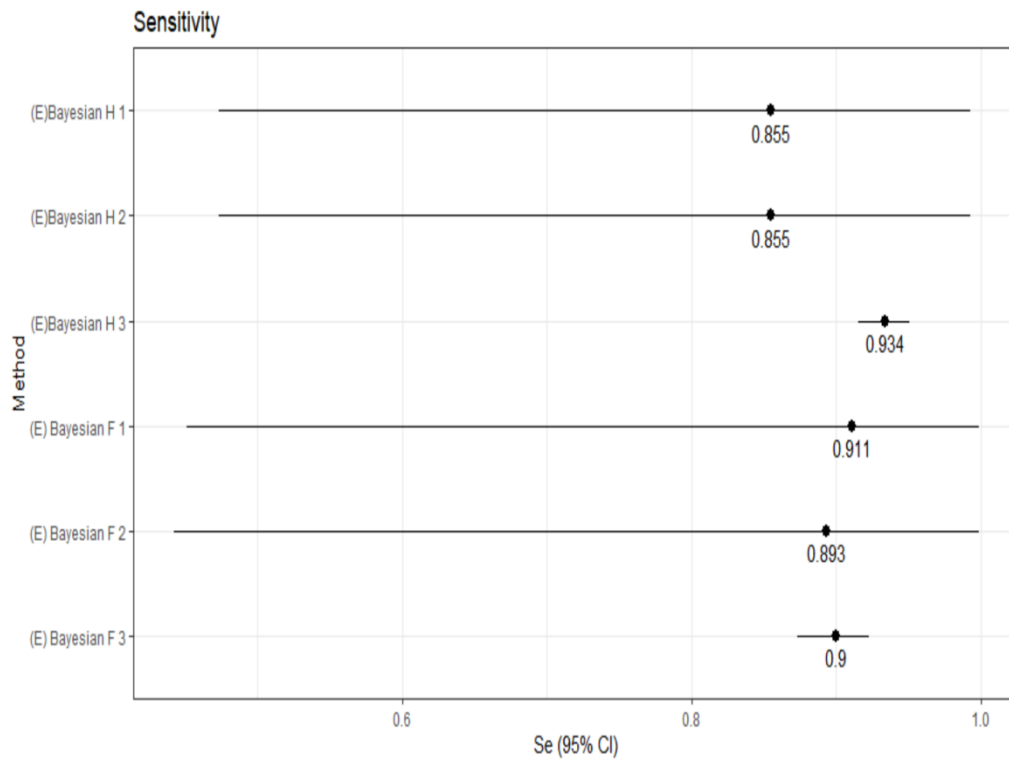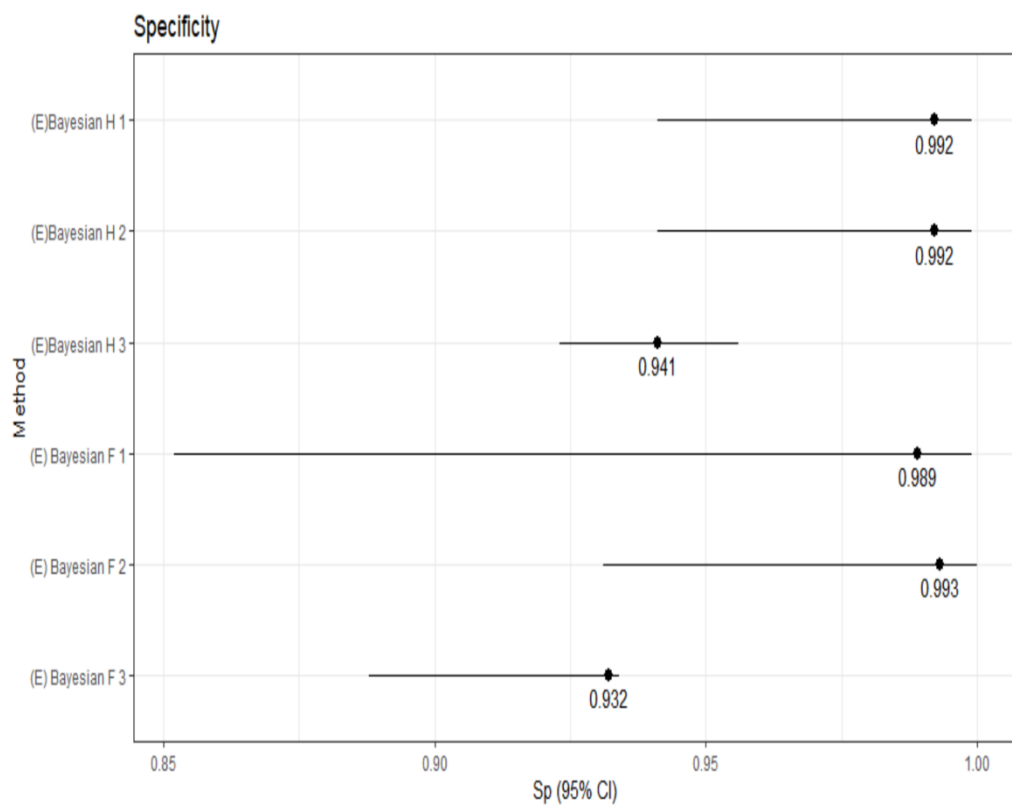

Supplement: Supplementary file 1 — Additional file 1 Figure legend: (E): excluding patients lost to follow-up; H: with respect to histology; F: with respect to follow-up; (1): non-informative beta priors for FNAC, informative prior distribution (Beta (172.55, 30.45)) for both Se and Sp of imaging; (2): non-informative beta priors for FNAC and for imaging with respect to follow-up; (3): informative priors for the Se and Sp of FNAC (beta(525,55.1) and beta(465,56)respectively), informative priors for imaging as used in (1) [file 12874_2022_1506_MOESM1_ESM.pdf]
